# Methodology optimizing SAGE library tag-to-gene mapping: application to *Leishmania*

**DOI:** 10.1186/1756-0500-5-74

**Published:** 2012-01-27

**Authors:** Sondos Smandi, Fatma Z Guerfali, Mohamed Farhat, Khadija Ben-Aissa, Dhafer Laouini, Lamia Guizani-Tabbane, Koussay Dellagi, Alia Benkahla

**Affiliations:** 1 Laboratoire d'Immuno-Pathologie, Vaccinologie et Génétique Moléculaire (LIVGM), WHO Collaborating Center for Research and Training in Leishmaniasis, Institut Pasteur de Tunis, 13 place Pasteur BP74 1002, Tunis, Tunisia

## Abstract

**Background:**

Leishmaniasis are widespread parasitic-diseases with an urgent need for more active and less toxic drugs and for effective vaccines. Understanding the biology of the parasite especially in the context of host parasite interaction is a crucial step towards such improvements in therapy and control. Several experimental approaches including SAGE (Serial analysis of gene expression) have been developed in order to investigate the parasite transcriptome organisation and plasticity. Usual SAGE tag-to-gene mapping techniques are inadequate because almost all tags are normally located in the 3'-UTR outside the CDS, whereas most information available for *Leishmania *transcripts is restricted to the CDS predictions. The aim of this work is to optimize a SAGE libraries tag-to-gene mapping technique and to show how this development improves the understanding of *Leishmania *transcriptome.

**Findings:**

The in silico method implemented herein was based on mapping the tags to *Leishmania *genome using BLAST then mapping the tags to their gene using a data-driven probability distribution. This optimized tag-to-gene mappings improved the knowledge of *Leishmania *genome structure and transcription. It allowed analyzing the expression of a maximal number of *Leishmania *genes, the delimitation of the 3' UTR of 478 genes and the identification of biological processes that are differentially modulated during the promastigote to amastigote differentiation.

**Conclusion:**

The developed method optimizes the assignment of SAGE tags in trypanosomatidae genomes as well as in any genome having polycistronic transcription and small intergenic regions.

## Background

*Leishmania*, the causative agent of leishmaniasis, is a protozoan parasite of the order Kinetoplastida. The *Leishmania major *genome is 33 Mb in size with a karyotype of 36 chromosomes. There are 911 RNA genes, 39 pseudogenes, 8272 protein coding genes of which 36% can be ascribed a putative function. The means of CDS and intergenic regions length are 1901 bp and 2045 bp, respectively [1].

*Leishmania *species exist in two distinct stages within the mammalian host they infect. Promastigotes, present in the sand fly insect vector, are inoculated to mammalian hosts, where they transform into amastigotes, a form adapted to survive within these mammalian host cells. The molecular events allowing the differentiation from promastigotes to amastigotes are still poorly understood. The post-transcriptional and/or post-translational regulation of genes involved in several biological processes is certainly important to adapt the parasite to survive in the harsh conditions of the parasitophorous vacuole and to circumvent the host's immune response. Hence, a systematic identification of these genes is necessary to understand the mechanisms underlying parasite intracellular survival.

Several gene expression experiments were performed on different *Leishmania *species using DNA, cDNA, oligonucleotides microarrays or SAGE technology [2-8]. Different studies performed at the transcriptomic level have focused, either on genes differentially expressed between promastigote and amastigote stages, or between distinct *Leishmania *species. Modulated genes encode for proteins with either hypothetical or unknown functions or for proteins with known function e.g. surface proteins, kinases, maintenance protein, metabolic enzymes, structural genes, transporters, and heat shock proteins.

SAGE is an approach that allows the rapid, quantitative, simultaneous and detailed analysis of thousands of transcripts [9], and is a powerful tool for the analysis of genome-wide gene expression without requiring knowledge of the gene content [10]. It was successfully used in a wide variety of organisms and applications including the elucidation of diseases [11], the detection of transcripts expressed at low levels [12] and the discovery of new genes [13]. Its output is a list of short sequence tags which size depend on the specific SAGE technology used. One of the critical steps using SAGE technique is the tag-to-gene mapping. Classical methods involve the mapping of any SAGE tag to the 3' most tag within each transcript. These methods either use known 3'UTR or artificially extend the 3'UTR of predicted genes or both. Unfortunately, these methods are not applicable for the study of organisms like *Leishmania *for which the full transcripts and the approximate length of their 3'UTR are unknown.

**Figure 1:**
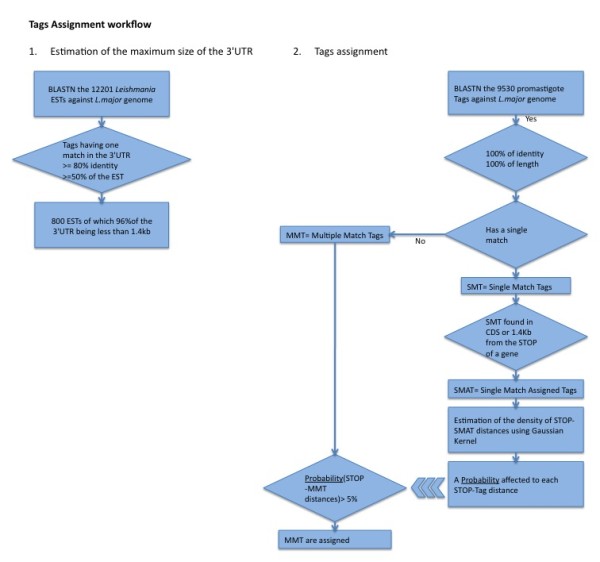
**Bioinformatics workflows: 1) Estimation of the maximum size of the 3'UTR**. 2) Tags assignment. The three arrows mean that the probability derived from the estimation of the density of STOP-SMAT distances were used to evaluate the STOP-MMT distances.

In a previous report [3] using SAGE, we have provided a large-scale gene expression profile of *Leishmania major *(*L. major*) promastigotes (Lm) and *Leishmania *infected Monocyte Derived Macrophages (MDM + Lm). The used SAGE technology produces 14nt cDNA tags. The tag-to-gene assignment technique was basic and the assignment restricted to the most abundant tags and to tags preferentially expressed by intra-macrophagic parasites. As a result several tags/genes were excluded from the analysis because they could not be assigned to their respective genes or because they had a steady expression. Considering this limitation, we present here a novel method that optimizes the tag-to-gene mapping process for all expressed tags. This method does not validate the SAGE libraries, it exploits the genomic sequence and gives different confidence values to each of the hits of a SAGE-tag in the genome. The confidence values were defined according to parameters obtained through the kernel density estimation (kde) [14] of a distribution of unambiguously assigned tags. A detailed workflow is shown in Figure 1.

The implemented technique allowed the assignment of a maximum number of SAGE-tags, and the evaluation of the expression of their transcripts and the delimitation of some 3'UTR. These results pushed a step forwards the initial analysis [3] by integrating the expression of a larger number of genes and extending the knowledge of *Leishmania *3'UTR. Additionally, we systematically characterized the function of genes differentially expressed in Lm versus the library generated at amastigote stage (MDM + Lm) [3].

## Results

**Figure 2:**
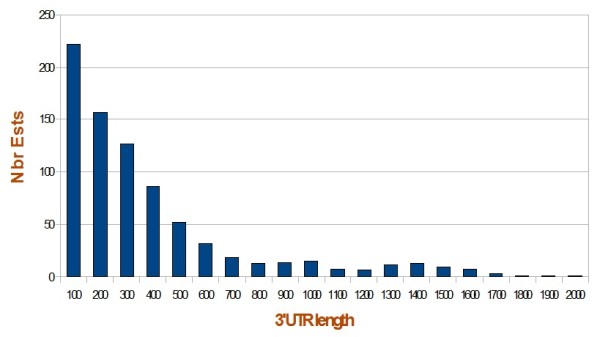
**Histogram illustrating the size distribution of the 3'UTR of 800 3'ESTs mapped on the *L. major *genome**. 573 ESTs overlapped with the CDS and with the 3'UTR of transcripts and 227 ESTs were in the 3'UTR of transcripts. 96% of the latter 3'UTR are less than 1.4 kb.

The main result of the present study is the development of a tag-to-gene assignment tool (R program available in the additional files). The developed program assigns a maximum number of SAGE-tags [3], to their respective genes. Multiple match tags (MMT) are the tags which assignment was the trickiest. The program uses a data-driven probability derived from the mapping of a set of single match assigned tags (SMAT), and evaluates the most probable tag-to-gene association of MMT. Results are available in Additional file 1. The limit 1.4 kb, maximal tolerated distance between the CDS and the end of the 3'UTR, was derived from the 800 mapped 3'ESTs available in Genbank on November 2010 (See Figure 2 and Materials and Methods - Estimation of the maximum size of the 3'UTR paragraph). Because 1.4 kb might be considered as being too short or too large, the authors have parameterized this length into the source R program leaving to the user the freedom to rerun the program with the distance that suits them best.

According to the method implemented for tags assignment, 7766 out of the 9530 unique tags were mapped on the parasite genome. Among these, 4168 tags were classified as single match tags (SMT) and 3598 as MMT. Among SMTs, 3171 were classified as SMAT and assigned to 2538 different genes. The 3598 MMT had 13617 mappings (on average 4 mappings/tag), and out of these, 908 tags were assigned to 832 different genes. In total, the implemented strategy was able to successfully assign 4079 tags (52.56% of total sequenced tags) to 3094 genes (37% of total genes) (Additional file 2).

Because gene expression is known to be driven from the 'sense' and 'antisense' strand (complementary) of DNA in *Leishmania *[15-17], we have used our SAGE data to predict the antisense gene transcription. From the 3171 SMT, 2449 were assigned to genes in the same direction ('sense') and 722 to genes in the opposite direction ('antisense'). From the 908 assigned MMT, 610 were sense and 298 antisense. In total, 2148 genes showed no antisense transcription and 470 no sense transcription, whereas 476 showed both a sense and an antisense transcription. This result estimates the rate of antisense genes as being higher than expected (~30%).

Among the 3094 identified genes, 772 were represented with different tags (275 with tags in the same direction, 21 with tags in the opposite direction and 476 with tags in both directions). 11 of these had significant expression changes (LmjF10.0090, LmjF15.0950, LmjF15.1203, LmjF24.2080, LmjF26.2220, LmjF29.1730, LmjF29.2370, LmjF34.2900, LmjF35.1890, LmjF36.3620 and LmjF36.6680); the direction of their expression change was the same according to the different tags counts. The list of these 11 genes is enriched in genes showing sense and antisense co-transcription (5/11) an observation that might reflect one of *Leishmania *post-transcriptional regulatory mechanisms.

Apart from the estimation of the expression of a maximum number of genes and the identification and the GO characterization of a maximum number of differentially expressed genes; the developed method allowed the delimitation of some 3'UTR.

### Functional characterization of differentially expressed genes

A large proportion of *L. major *genes are differentially expressed between promastigote and amastigote stages [3]. Indeed, a total of 304 genes showed a significant differential expression between Lm and MDM + Lm, indicating that they might play a role in the parasite differentiation and/or in *Leishmania *virulence. Among these 304 genes, 189 and 115 genes were preferentially expressed by amastigotes and promastigotes respectively. The functional analysis of these genes using GOTermMapper and GOTermFinder revealed that 102 genes could be classified according to their biological processes, whereas 202 genes had no functional annotation (Additional file 3 and Additional file 4). KEGG pathways [18] associated to these genes (Additional file 4) revealed that 65 genes are Involved in known pathways. This result gives an idea about the *Leishmania *processes and pathways that are preferentially activated during the different stages.

### 3'UTR characterization

PRED-A-TERM allowed the prediction of 1581 3'UTR extremities (337 were located between the STOP and the tag; 452 were located between the tag and the following CATG; 648 between the following CATG and the start of the following gene; and 144 after the start of the following gene). Following the approach detailed in the paragraph 3'UTR characterization (in the Materials and Methods), we were able to validate the PRED-A-TERM predictions for 478 different *L. major *genes (Additional file 5): These were grouped into 452 predictions validated using the tags data and 26 using the EST data.

## Discussion

To assign the maximum number of tags to their respective genes in a SAGE library constructed from metacyclic *Leishmania *promastigotes [3], we developed a method that evaluates the most likely tag-to-gene association of MMT. The implemented method allowed the assignment of a maximum number of SAGE tags, without arbitrarily fixing the size of the 3'UTR as it was previously done for the assignment of Arabidopsis tags [19,20].

Unlike the method presented by Malig and colleagues [21], the one developed here dealt with experimental tags and provided an estimation of the confidence on a tag-to-gene assignment. Malig and colleagues method [21] dealt with virtual SAGE tags and evaluated the tag-to-gene assignment intuitively. We also preferred to use what we have learned from the frequency distribution of the distance of SMAT from the STOP of the associated gene rather than extending the 3'UTR by a fixed number of nucleotides as in Pleasance and colleagues method [22].

SAGE technology is being routinely used to detect antisense transcription [23]. Such prediction has the same value as the prediction of sense transcription. The fact that antisense is being more common than expected (~30%) is presumed since gene regulation in *Leishmania *is driven through post-transcriptional mechanisms likely modulated through antisense transcription [24].

In our previous study [3], the purpose was to capture strong functional signals emitted during the differentiation from promastigotes to amastigotes. The tag-to-gene assignment was basic and not generalized to all tags. The assignment was restricted to the most abundant tags (1.1% of the total number of unique tags) and to tags preferentially expressed by intra-macrophagic parasites. The optimization of the tag-to-gene assignment procedure herein allowed the identification of a maximum number of expressed and differentially expressed genes. The consequence of the latter allowed the identification of 76 additional genes preferentially expressed by intra-macrophagic amastigotes [3].

Several statistical tests are available to evaluate differential expression in SAGE libraries. The comparison of the output of the used test [25] to that of other tests (Student's test [26] and Shapiro-Wilk test [27]) showed, as expected, no significant differences. Therefore, the used statistics should not impact on the interpretation of the results. However, it has to be noted that libraries of a size lower than 120000 (the size recommended for SAGE experiments [28]), could under-estimate the number of expressed and differentially expressed genes which could impact on the interpretation of the results. This might explain why some genes are not observed as being constitutively expressed as previously reported [6].

Compared to our previous study, the functional GO characterization was done for all differentially expressed tags. GOTermMapper, GoTermFinder and KEGG pathways annotation indicated that the list of modulated genes is enriched in genes which end products are involved in RNA 'translation', inferring that translation related processes are affected by the intracellular development of the parasite. This result agreed with those reported by McNicoll and colleagues [8] who suggested that protein translation is affected during promastigote to amastigote differentiation (a weak correlation was observed between the transcriptome and the proteome levels at the amastigote stage and no correlation at the promastigote stage) and that translational and post-translational mechanisms are important for controlling gene expression.

Clayton and Shapira show in 2008 [29] that the parasite uses a polycistronic transcriptional approach and that mRNA abundance is regulated by post-transcriptional mechanisms driven through elements located in the 3' untranslated region (3'UTR) [8,29]. Therefore, to be properly interpreted, transcriptome data would need the development of tools that allow the study of the post-transcriptional regulatory elements located in the 3'UTR. The first step towards this development is a better delimitation of the 3'UTRs of known genes.

As the 478 3'UTR extremity predictions were supported by experimental data (i.e.: *Leishmania *3'EST and/or *L. major *tags), they could be considered as true predictions and the predicted 3'UTR could be used, in our opinion, straight away for the investigation of post-transcriptional regulatory motifs. Additionally, the 3'UTR extremity predictions located between the CATG following a tag and the start of the following gene can be valid and would have to be validated through the sequencing of the corresponding transcript. Predictions located elsewhere should be considered as false positives unless they correspond to overlapping genes which existence is to be proven in *Leishmania*.

The presented method was not able to assign 5451 out of 9530 tags, among which 1764 were not mapped to *L. major *genome, which could be due to several reasons including tags sequencing errors (false positives), small size of the tags (14 nt), or polymorphic variations between the Friedlin strain used for the genome sequencing and the strain used to construct our SAGE library, or the presence of microbial component. Some unassigned tags could also belong to new protein or non-protein coding genes, absent from the GeneDB catalogue. These tags would gain to be further investigated to obtain an exhaustive *L. major *genes catalogue. It has also long been known that non-coding RNAs are relatively common in trypanosomatids, and represent relatively stable processing products from polycistronic transcripts [30]. Since the existence of non-protein coding genes was not considered because of the absence of a dedicated catalogue, this has probably contributed to the raising of the mis- and non-assigned tags rate and implied that a proportion of the remaining non-assigned tags (the SMT mapping between two genes and some MMT) could belong to this class of genes. The lack of exhaustive knowledge about the actual full transcripts catalogue could also generate erroneous tag-to-gene associations of multiple match tags. Polymorphism within the *L. major *genus and the tags sequencing errors were additional parameters that can increase these rates.

The *Leishmania *genome was characterized by the presence of duplicated segments containing large gene families [1]. MMT mappings into members of the same gene family were difficult to assign and often remained unassigned. A complementary approach would be considering the assignment of these tags to conserved gene families' rather than individual genes.

## Conclusions

In the present study, we developed a novel approach for a better assignment of the SAGE tags to their genes for all organisms having polycistronic transcription and small intergenic regions. It expanded classical tag assignment methods to a method that does not require any extended knowledge concerning the 3'UTR. The implicit consequence of optimizing the assignment process was to approximate sense and antisense tag expression and to identify a maximum number of Leishmania genes that are differentially expressed. It should however worthy to note that all these assignments need to be validated experimentally.

The comparison of gene expression profile between promastigote and amastigote stage and the systematic GO classification of modulated proteins allowed the identification of the key biological processes that are modulated during differentiation from promastigotes to amastigotes. An exhaustive functional annotation of the genes involved in these processes would be helpful in understanding the mechanisms of intracellular parasite differentiation and in the identification of new drug target proteins.

In addition, a new approach indicating the most likely 3'UTR extremity of *Leishmania *genes is presented. This approach is based on the assessment of predicted *Leishmania *3'UTR mRNA extremity by supporting biological evidences (Lm tags, *Leishmania *Expressed Sequence Tags and one prediction tool [31]).

## Materials and methods

### Generation of SAGE libraries

SAGE libraries were previously generated by our group and corresponded to *L. major *GLC94 metacyclic extracellular promastigotes (Lm) and to *L. major *GLC94 in their intracellular form within infected macrophages (MDM + Lm). All details about the parasite culture and preparation, the RNA isolation, and the SAGE library construction and its computer-based analysis were described by Guerfali and colleagues [3]. Lm was obtained from purified metacyclic *L. major *promastigotes and contained a total of 33,906 SAGE-tags corresponding to a non-redundant set of 9,530 different tags. MDM + Lm was sequenced from *L. major*-infected macrophages and contains 57,514 tags corresponding to a non-redundant set of 24,418 tags. Both libraries associated each tag to its frequency.

### Tags assignment

#### (a) Estimation of the maximum size of the 3'UTR

The 3'UTR of a given gene corresponds to the 3' sequence of a mature transcript that is not translated. The size of 3'UTR of *Leishmania *genes is relatively large and variable. Prior reports estimated, for example, the 3'UTR of HSP70 [32] and HSP83 [33] to 1063 and 886nt, respectively. Moreover, 12201 ESTs of *Leishmania *available on Genbank were blasted against *L. major *genome. The 800 3'ESTs having a single match and mapping in the 3'UTR of a known gene, with ≥ 80% identity and over ≥ 50% of their length, were identified and considered as ESTs with known 3'UTR. Out of these, 129 3'ESTs are *L. major *ESTs. The size varied from 1 to more than 2000 nt; 96% of the 3'UTRs being less than 1.4 kb (see Figure 2). This distance will be considered as the maximal tolerated distance between the CDS and the end of the 3'UTR. The list and the description of these 3'UTR is available in Additional file 6. 1.4 kb is used in the assignment approach paragraph as maximum length of a 3'UTR.

#### (b) Assignment approach

The non-redundant set of 9,530 promastigote SAGE-tags were mapped to *L. major *genome by blasting [34] the 14 nt tag sequences against the latest release of the parasite genome available and downloaded from GeneDB [35] on December 8, 2006. The distances between matching tags (100% of identity over 100% of the length) and the STOP codon of the nearest gene were retrieved.

Assigning SMT to the closest predicted CDS is quite natural. The closest CDS being the one located less than 1.4 kb (maximal tolerated distance between the CDS and the end of the 3'UTR), each SMT found within the coding sequence or 1.4 kb downstream the STOP codon of a given gene, was assigned to that particular gene. Accordingly, they were classified as SMAT. Tags not verifying these conditions (i.e. SMT within more than 1.4 kb away from any gene or MMT) were kept for further investigations.

A Gaussian Kernel Density Estimation approach [14] was then used to estimate the density of SMAT distances; the estimation function being:

$${\hat{f}}_{h}\left(x\right)=\frac{1}{Nh}\overset{N}{\underset{i=1}{\sum }}K\left(\frac{x-x_{i}}{h}\right)$$

where N is the size of SMAT dataset, x is the set of breakpoints, h is the bandwidth (smoothing parameter), and K is the standard Gaussian function with mean zero and variance 1:

$$K\;\left(x\right)=\frac{1}{\sqrt{2p}}e^{-\frac{1}{2}x^{2}}$$

The X-axis was later split into 50 classes, *(x_i_)_i ≤ _*_50_, each one corresponding to 41 nt (41 = (max of x - min of x)/50), and a probability *P_j _*was affected to each class j.

*P_j _*being equal to the difference between two consecutive Cumulative Density Functions (CDF):

$$P_{j}=CDF\left[j+1\right]-CDF\left[j\right]\;\left(j=1,\ldots ,49\right)$$

where

$$CDF\left(jv\right)=\overset{x_{j}}{\underset{x}{\int }}{\hat{f}}_{h}\left(t\right)dt$$

**Figure 3:**
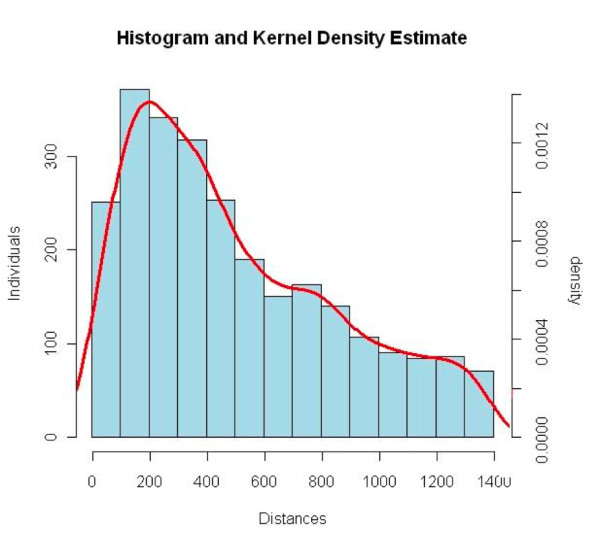
**Gaussian kernel density estimation of the assigned tags**. The x-axis represents the size of the segment STOP-tag. The right and left y-axis do not correspond to the same curve and have different scales. The right y-axis is for the red curve and represents the density of SMAT distances. The left y-axis is for the histogram and represents the tags count in the appropriate segment STOP-tag. Linear binning is used to obtain the bin counts (500) on the x-axis.

A plot of this probability is shown in Figure 3. To assess the most probable tag-to-gene association, these probabilities were attributed to the distances between the mappings of the MMT and the STOP of the nearest gene. The tag-to-gene association having a probability higher than 5%, were selected.

#### (c) Differentiate assigned 'sense' and 'antisense' tags

SAGE tags being directionally reliable short cDNA sequences [36], we have defined 'sense tags' as tags mapping to a given gene and 'antisense tags' as tags mapping to the reverse complement of a given gene.

### Functional characterization of differentially expressed genes

The 697 tags listed by Guerfali and colleagues [3] (Additional file 7), present in the MDM + Lm and Lm and absent in other human libraries, and differentially expressed, were reloaded. Of these tags, 420 were found to be preferentially expressed by amastigotes, and 277 by promastigotes. Genes associated to these tags were considered as differentially expressed.

The biological process(es) to which the differentially expressed genes belong to were characterized using GOTermMapper (http://go.princeton.edu/cgi-bin/GOTermMapper) and GOTermFinder [37].

### 3'UTR characterization

SAGE libraries were generated using polyA + RNA and converted to cDNA. The latter were cleaved with the NlaIII enzyme after the first CATG encountered and the 3'-terminal cDNA fragment were bound to streptavidin-coated beads. After concatemerization, these SAGE tags were sequenced. The sequenced tags should correspond to the first 14 nt fragment containing a CATG at the most 3'-end position of the mRNA (unless the mRNA contains a second polyA stretch). Departing from this observation, the true positive PRED-A-TERM polyA predictions [31] of all genes with an assigned tag (3094 genes) should be mapping in the 3'UTR of that gene and between the assigned tag and the following CATG.

As recommended by Smith and colleagues [31], PRED-A-TERM was run on the intergenic sequences of all genes with an assigned tag (3094 genes), plus the last 800 nt of the upstream gene, and the first 800 nt of the downstream gene. All predictions mapping in the 3'UTR of the upstream gene and between the corresponding tag and the following CATG or less than 100 nt away from the end of a mapped EST, were considered as a likely true polyA region prediction.

## Abbreviations

SAGE: Serial analysis of gene expression; UTR: UnTranslated Region; CDS: Coding Sequence; DNA: Deoxyribonucleic Acid; cDNA: Complementary Deoxyribonucleic acid; Lm: Promastigotes; MDM + Lm: Leishmania infected Monocyte Derived Macrophages; kde: Kernel Density Estimation; MMT: Multiple Match Tags; SMAT: Single Match Assigned Tags; SMT: Single Match Tags; KEGG: Kyoto Encyclopedia of Genes and Genomes; ORF: Open Reading Frame; EST: Expressed Sequence Tag; mRNA: Messanger Ribonucleic Acid; nt: Nucleotide; Kb: KiloBase; PolyA: PolyAdenosine

## Competing interests

The authors declare that they have no competing interests.

## Authors' contributions

Sondos Smandi did all the analyses and participated in the writing. Mohamed Farhat did implement the statistical analysis. Alia Benkahla did coordinate all the analyses and the writing. Fatma Guerfali, Dhafer Laouini and Lamia Guizani-Tabbane did participate to the analysis of the results and the writing. Koussay Dellagi did initiate the project and participate to the writing. All authors have read and approved the final manuscript.

## Supplementary Material

Additional file 1 **The probability attributed to the distance between the mappings of the MMT and the stop of the nearest gene**. Click here for file

Additional file 2 **List of assigned tags from library Lm and their corresponding genes**. Click here for file

Additional file 3 **Distribution of *L. major *differentially expressed genes according to Genes Ontology (GO) biological process categories**. The majority of genes code for proteins with no GO category. The metabolic process category is composed of genes involved in different processes (72 genes in primary metabolic process; 62 genes in protein metabolic process; 48 genes in biosynthetic process; 47 genes in the translation; 13 genes in transcription and nucleic acid metabolic process; 2 genes in lipid metabolic process). GOTermFinder estimates that the list of these genes is enriched in translation related proteins. Click here for file

Additional file 4 **GoTermMapper biological process classification of modulated genes**. Click here for file

Additional file 5 **Genes' 3'UTR extremity predictions (452 using PRED-A-TERM and tags, and 26 using PRED-A-TERM and EST)**. Click here for file

Additional file 6 **Description of the 3'UTR of 800 3'EST**. Click here for file

Additional file 7 **List of the modulated tags listed by Guerfali and colleagues (2008)**. Click here for file
