# Supplementary figures and images for: Methodology optimizing SAGE library tag-to-gene mapping: application to Leishmania
Source: BMC Res Notes. 2012 Jan 27;5:74. doi: 10.1186/1756-0500-5-74 (PMC3292834; doi:10.1186/1756-0500-5-74)

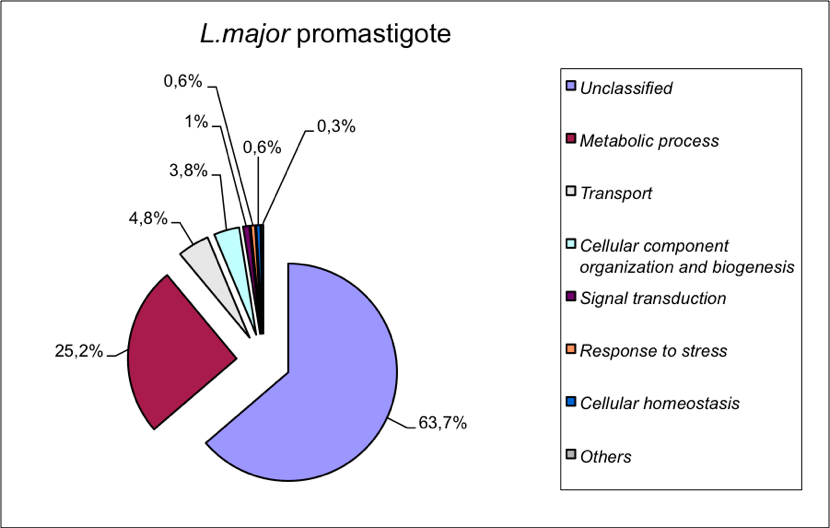

Supplement: Additional file 3 — Distribution of L. major differentially expressed genes according to Genes Ontology (GO) biological process categories. The majority of genes code for proteins with no GO category. The metabolic process category is composed of genes involved in different processes (72 genes in primary metabolic process; 62 genes in protein metabolic process; 48 genes in biosynthetic process; 47 genes in the translation; 13 genes in transcription and nucleic acid metabolic process; 2 genes in lipid metabolic process). GOTermFinder estimates that the list of these genes is enriched in translation related proteins. [file 1756-0500-5-74-S3.JPEG]
